# Identification of Small Molecule Inhibitors against *Staphylococcus aureus* Dihydroorotase via HTS

**DOI:** 10.3390/ijms22189984

**Published:** 2021-09-15

**Authors:** Amy J. Rice, Russell P. Pesavento, Jinhong Ren, Isoo Youn, Youngjin Kwon, Kassapa Ellepola, Chun-Tao Che, Michael E. Johnson, Hyun Lee

**Affiliations:** 1 Center for Biomolecular Sciences, College of Pharmacy, University of Illinois at Chicago, Chicago, IL 60607, USA; arice1023@gmail.com (A.J.R.); rpesaven@uic.edu (R.P.P.); jhren@uic.edu (J.R.); ykwon35@uic.edu (Y.K.); 2 Department of Pharmaceutical Sciences, College of Pharmacy, University of Illinois at Chicago, Chicago, IL 60612, USA; iyoun2@uic.edu (I.Y.); chect@uic.edu (C.-T.C.); 3 Department of Oral Biology, College of Dentistry, University of Illinois at Chicago, Chicago, IL 60607, USA; kassapa@uic.edu; 4 Biophysics Core at the Research Resource Center, University of Illinois at Chicago, Chicago, IL 60607, USA

**Keywords:** *S. aureus*, antimicrobial, dihydroorotase, Class I DHOase, inhibitors

## Abstract

Drug-resistant *Staphylococcus aureus* is an imminent threat to public health, increasing the importance of drug discovery utilizing unexplored bacterial pathways and enzyme targets. *De novo* pyrimidine biosynthesis is a specialized, highly conserved pathway implicated in both the survival and virulence of several clinically relevant pathogens. Class I dihydroorotase (DHOase) is a separate and distinct enzyme present in gram positive bacteria (i.e., *S. aureus*, *B. anthracis*) that converts carbamoyl-aspartate (Ca-asp) to dihydroorotate (DHO)—an integral step in the *de novo* pyrimidine biosynthesis pathway. This study sets forth a high-throughput screening (HTS) of 3000 fragment compounds by a colorimetry-based enzymatic assay as a primary screen, identifying small molecule inhibitors of *S. aureus* DHOase (*Sa*DHOase), followed by hit validation with a direct binding analysis using surface plasmon resonance (SPR). Competition SPR studies of six hit compounds and eight additional analogs with the substrate Ca-asp determined the best compound to be a competitive inhibitor with a *K*_D_ value of 11 µM, which is 10-fold tighter than Ca-asp. Preliminary structure–activity relationship (SAR) provides the foundation for further structure-based antimicrobial inhibitor design against *S. aureus*.

## 1. Introduction

*Staphylococcus aureus* is commonly associated with several common forms of infection, including impetigo, cellulitis, atopic dermatitis [1] and osteomyelitis [2]. Although treatment of these infections often rely upon β-lactam (e.g., methicillin) and/or glycopeptide-based antimicrobial agents (e.g., vancomycin) [3,4], their continued use has led to poor efficacy against emerging resistant forms, such as methicillin-resistant *S. aureus* (MRSA). Such resistance, combined with sustained biofilm formation, has contributed to the development of more serious infections, including bacteremia [5], bronchiectasis [6] and bacterial endocarditis [7,8]. To combat both the widespread pathology and emerging resistance of *S. aureus*, new pharmacological agents with novel modes of inhibition should be continually investigated.

**Figure 1:**
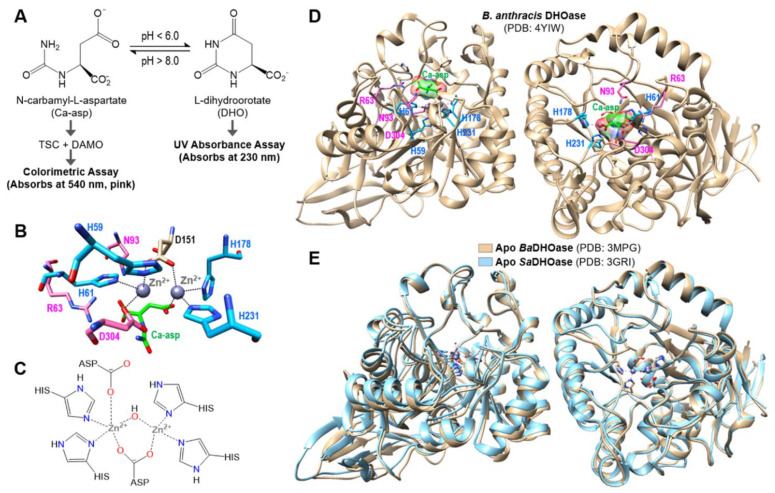
Background information. (**A**) The pH-dependent, reversible cyclization of N-carbamyl-L-aspartate (Ca-asp) to L-dihydroorotate (DHO) catalyzed by DHOase. Two assays to detect Ca-asp and DHO. (**B**) Dimeric structure of *Bacillus anthracis* DHOase complex with Ca-asp (PDB: 4YIW). The Ca-asp and three catalytic residues (R63, N93 and D304) are shown in green and pink, respectively. Four zinc-binding histidines are in blue. (**C**) The active site of Class I DHOase displaying two inequivalent Zn (II) ions. (**D**) The active site of *B. anthracis* DHOase with Ca-asp bound (PDB: 4YIW). The Ca-asp and three catalytic residues (R63, N93 and D304) are shown in green and pink, respectively. Four zinc-binding histidines are in blue. (**E**) Overlaid structures of apo DHOases from *B. anthracis* and *S. aureus*. Apo *Ba*DHOase and *Sa*DHOase are shown in tan and cyan, respectively.

Dihydroorotase (DHOase) is the third enzyme utilized in the ubiquitous *de novo* pyrimidine biosynthesis pathway responsible for nucleic acid production. DHOase catalyzes the pH dependent, reversible hydrolytic cyclization of N-carbamyl-L-aspartate (Ca-asp) to L-dihydroorotate (DHO) (Figure 1A). DHOase has been historically divided into three classes separated by host phylogeny and key structural differences. Class I DHOase is found in gram-positive bacteria (e.g., *S. aureus*, *Bacillus anthracis*), mold and insects and maintains a highly conserved active site defined by three catalytic residues (Arg, Asn and Asp) and two inequivalent Zn(II) ions coordinated with four histidines (Figure 1B,C). Fungi and gram-negative bacteria, such as *Escherichia coli*, belong to Class II DHOase. Very recent structural studies reported *Saccharomyces cerevisiae* DHOase complex structures with 5-fluorouracil (5-FU) and 5-aminouracil (5-AU) [9]. There are three structures solved by X-ray crystallography for the Class I DHOases to date, two of which are *B. anthracis* DHOase (*Ba*DHOase) structures, apo (PDB:3MPG) and substrate Ca-asp bound (Figure 1D). The third one is an apo *S. aureus* DHOase (*Sa*DHOase), and it overlays well with the apo *Ba*DHOase structure shown in Figure 1E [10]. DHOase is a separate and distinct enzyme in the *de novo* pyrimidine biosynthesis pathway. This is in direct contrast to human DHOase (Class III), wherein the enzyme is part of a larger, multifunctional enzyme. The segregation of Class I DHOase, as compared to Class III DHOase, allows for potential specificity in selectively inhibiting bacterial DHOase over its mammalian counterpart [11]. Overall, the above properties make Class I DHOase a potential target in designing novel antimicrobial agents.

Although Class I DHOase has been suggested to be a potential target for antimicrobial agents [11] for several years, few reports have demonstrated its enzymatic inhibition in vitro. Johnson and co-workers utilized high throughput screening (HTS) to first demonstrate the in vitro inhibition of Class I DHOase (*B. anthracis*) [10]. A substituted indole molecular fragment was found to be the most active moiety in inhibiting *Ba*DHOase in vitro. The current study expands upon our earlier report by identifying a novel molecular fragment observed to inhibit *S. aureus* DHOase (i.e., *Sa*DHOase, Newman strain) [12] in vitro. HTS via a previously developed colorimetric assay [13], surface plasmon resonance (SPR) assays and docking studies provide support for a molecular fragment found to inhibit *Sa*DHOase in vitro.

## 2. Results

### 2.1. Preparation, Purification and Activity of SaDHOase

The full-length SaDHOase was expressed and purified following the cloning of the SaDHO gene according to previously published methods [12]. Purified SaDHOase enzyme activity was measured and confirmed by two methods, one by observing the production of Ca-asp (reverse reaction) at a UV-Vis absorption of 540 nm (TSC-DAMO method) and the other with continuously monitoring reduction of DHO at 230 nm (Figure 1A). Enzyme rates were calculated using the measured slopes, path length of 0.44 cm and the extinction coefficient (ε) of DHO at 230 nm, 1.17 mM^−1^ cm^−1^ [14].

### 2.2. High-Throughput Screening and Secondary Confirmation of SaDHOase Inhibitors

**Figure 2:**
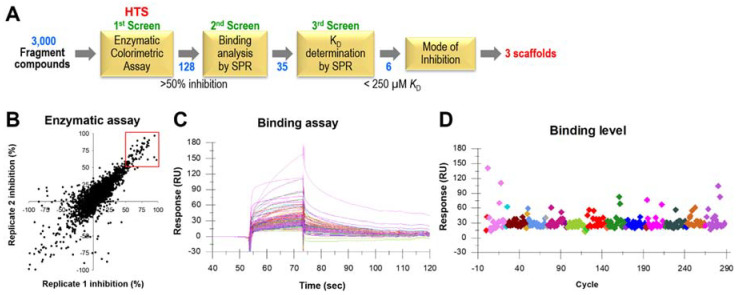
High-throughput screening results of SaDHOase with Chembridge fragment library. (**A**) Schematic representation of overall HTS and hit validation process. (**B**) Replicate plot of the primary HTS of 3000 fragment compounds from specially selected Chembridge by enzymatic assay, resulting in 128 hits highlighted in the red box. (**C**) Overlaid surface plasmon resonance (SPR) sensorgrams of 128 hit compounds directly binding to the immobilized SaDHOase at a single concentration (400 µM). (**D**) Binding level at 10 s before injection ended after zero concentration signal subtraction.

A high-throughput screen of the Chembridge fragment library, consisting of 3000 compounds was performed, against SaDHOase using the optimized enzymatic colorimetric assay previously described [13]. The overall HTS and follow-up studies are illustrated in Figure 2A. Any fragments that showed >50% inhibition of SaDHOase enzyme activity (red square in Figure 2B) compared to the control were selected as hits, resulting in 128 hits (Figure 2A). SPR provided a secondary direct binding assay to select hits that bind to SaDHOase and rapidly eliminate those that may be false positives by only affecting color development or inhibiting SaDHOase enzyme activity by denaturation or non-specific binding. This was done by using two concentrations of the hit compounds (65 µM and 200 µM), then selecting fragments that showed an increased binding level at the higher concentration. The substrate, Ca-asp, was used as a control for comparison of binding levels. Compounds that bind to SaDHOase showed a reasonable increase in binding level, as indicated by the increased response units at the higher concentrations, while compounds that were false positives showed no difference. Compounds with unreasonably high binding responses were also eliminated since it indicates a non-specific multi-site binding behavior. SPR screening of 128 hit compounds (Figure 2C,D) resulted in 35 compounds with confirmatory binding to the SaDHOase, but it does not discriminate between binding in the active site or allosteric sites.

### 2.3. Binding Affinity of Selected Hits and Catalytic Site Binders

**Figure 3:**
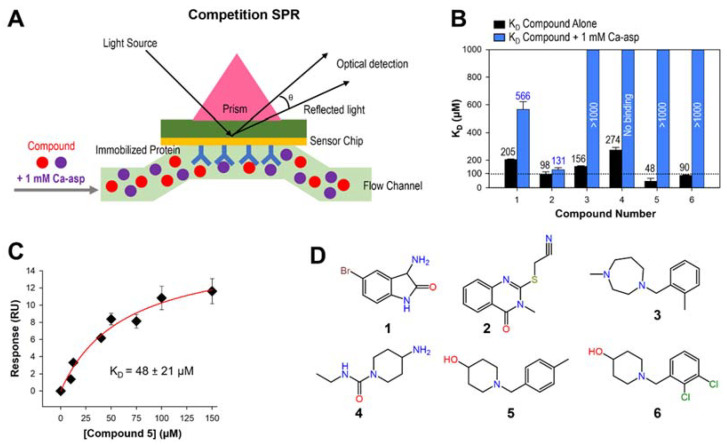
Binding affinity determination and catalytic site binders. (**A**) The schematic diagram of competition SPR with 1 mM substrate Ca-asp. (**B**) Bar graphs of the dissociation equilibrium constants (*K*_D_) value of 6 hit compounds. *K*_D_ values were compared in the presence (orange) and absence (black) of 1 mM substrate Ca-asp as a competitor. Bars that reached the top of the graph represent *K*_D_ values of >1000 µM wherein the enzyme did not reach saturation, so *K*_D_ was unable to be determined. (**C**) 1:1 Steady-state affinity fitting curve of the compound **5**. Determined *K*_D_ value of compound **5** was 48 ± 21 µM. (**D**) Chemical structures of six confirmed hit compounds.

Dose-response direct binding analysis by SPR study utilizing the 35 compounds ranging from 12.5 µM to 400 µM in 2-fold dilutions was used to eliminate non-specific binders and determine the dissociation equilibrium constant, *K*_D_. The *K*_D_ was determined using the response units during the equilibration phase of each concentration of the compound. Non-specific, or promiscuous, binders were identified as those that did not show a saturable curve when fit to the steady-state affinity model of the BiaEvaluation software. The exclusion of promiscuous binders resulted in 6 compounds for which the *K*_D_ was able to be determined, with values ranging from 48 to 274 µM (Figure 3B). In order to determine the mechanism of action, competition SPR studies were done in the presence of high concentration of the substrate Ca-asp (*K*_M_ = 110 µM) (Figure 3A). Dose-response curve of the tightest binder (**5**) is shown as an example (Figure 3C). Of the 6 compounds displaying saturable *Sa*DHOase binding curves, four compounds (**3**–**6**) displayed competitive binding in the presence of the substrate 1 mM Ca-asp (Figure 3B). Binding affinity of compound **1** became weaker ~2.8-fold in the presence of 1 mM Ca-asp, indicating it does compete with Ca-asp at least in part for the similar binding site. On the other hand, the *K*_D_ value of compound **2** was not affected by Ca-asp presence, suggesting it binds somewhere other than catalytic site. Two best active site binders, **5** and **6**, showed similar range-binding affinities (e.g., 48 µM and 90 µM, respectively), and both contain the 1-benzylpiperidine-4-ol scaffold (Figure 3D) which was further explored for activity below.

### 2.4. Preliminary Structure–Activity Relationship (SAR)

**Figure 4:**
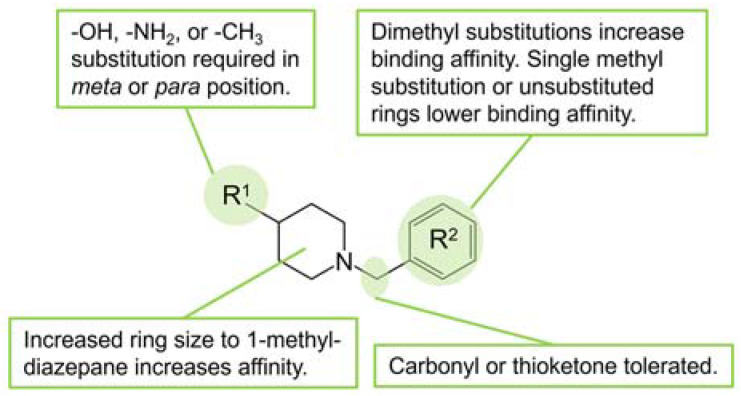
Structure–activity relationships (SAR) based on *K*_D_ values of analogs determined by SPR.

The similar binding kinetics and structural similarities in related compounds **5** and **6** suggested a possible structure–activity relationship. Eight commercially available analogs containing the core structural motif (1-benzylpiperidine-4-ol) conserved in compounds **5** and **6** were explored for their ability to bind to *Sa*DHOase, observable via SPR analysis. Since polar group substituents (dichloro) at the 2,3 positions in compound **6** resulted in a weaker *K*_D_, hydrophobic substitution was the initial focus of this limited study. Substitution with either -CF_3_ (**12**) or -CH_2_CH_3_ (**9**) at the 4-benzyl position alone results in no increase or significantly reduced binding affinity as compared to compound **5**. Further, single alkyl 3-benzyl substitution (**10**, **11**) eliminates all bonding of the 1-benzylpiperidin-4-ol moiety. The most potent molecule screened in this study was compound **8**, with a dimethyl substitution in the 2,5 positions (11 ± 5, *K*_D_), while single methyl substitution (**7**) did not increase binding to the enzyme (Table 1). A substituent is required on the piperidine ring with the para position having lower *K*_D_ values compared to the *meta* position as in compound **13** and **14**. A preliminary SAR map based on the hit compounds and their analogs was developed with the effects on *K*_D_ values (Figure 4). This preliminary SAR provides information for further structure expansion in developing inhibitors with high affinity for the *Sa*DHOase target.

The compounds for SAR study were further analyzed for quality control (QC) and purity assessment, and the procedures and QC results are shown in the Supplementary Materials.

### 2.5. Docking Studies

**Figure 5:**
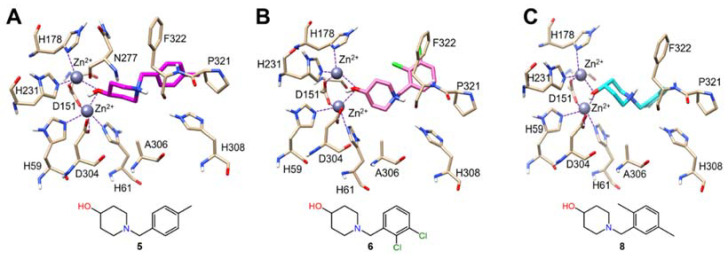
Predicted intermolecular interactions between *Ba*DHOase and compounds **5**, **6** and **8** from molecular docking. (**A**) Compound **5**, (**B**) **6** and (**C**) **8** are shown in the active site in magenta, pink and cyan, respectively. Intermolecular interactions are indicated with a dashed line to each Zn(II) ion.

Only three X-ray structures of Class I DHOases have been reported to date: one apo (PDB:3MPG) and one substrate bound (PDB:4YIW) structure of *Ba*DHOase, in addition to one apo *Sa*DHOase (PDB:3GRI) [10]. In both *Ba*DHOase structures, the active site was found to contain two inequivalent Zn(II) ions while the active site of apo *Sa*DHOase displayed only one Zn(II) ion in the active site. Because *Sa*DHOase maintains a high sequence identity (63%) with *Ba*DHOase, it is conceivable *Sa*DHOase may bind two Zn(II) ions under different crystallization conditions. As such, we opted to carry out the docking studies of **5**, **6** and **8** utilizing the *Ba*DHOase. The predicted binding poses for compounds **5**, **6** and **8** within the active site of *Ba*DHOase are shown below (Figure 5A–C). Compounds **5**, **6** and **8** formed *μ*-2 bridging interactions between the 4-OH group of the molecular fragment 1-benzylpiperidine-4-ol and α and β Zn(II) ions in the active site of *Ba*DHOase. Further, there is evidence of hydrophobic interactions stabilizing the 1-benzylpiperidine-4-ol fragment with surrounding residues: N277, A306, P321, F322 and H308 near the active site of *Ba*DHOase. Although metalloproteins are commonly inhibited by strongly bound chelating groups, hydrophobic interactions with surrounding residues are known to increase binding affinity [15].

## 3. Materials and Methods

### 3.1. Preparation and Purification of SaDHOase

The DHOase gene from *S. aureus* was cloned into a pET/SUMO vector with an HRV-protease cleavage site and purified as previously described [12]. In brief, the recombinant plasmid was transformed into BL21 (DE3) cells and grown in Luria–Bertani (LB) media with kanamycin (50 µg/mL) at 37 °C while shaking at 220 rpm until the OD_600_ reached 0.6, when it was induced with 0.5 mM IPTG and incubated for an additional 16 h at 25 °C before harvesting. The cell pellet was resuspended and lysed by sonication in lysis buffer (50 mM Tris, pH 8.0, 500 mM NaCl, 20 mM imidazole5 mM β-MCE, 1 mg/mL lysozyme, 1% Triton X-100 and 0.025 mg/mL DNase I). A HisTrap HP column was used to purify the histidine-tagged protein using a stepwise gradient of elution buffer (50 mM Tris, pH 8.0, 500 mM NaCl, 500 mM imidazole and 5 mM β-MCE) with either an AKTA purifier or AKTAxpress FPLC system. The histidine-HRV tag was removed by incubating the eluted protein with 1 unit/100 µg protein of HRV 3C protease in dialysis buffer (50 mM Tris, pH 7.5, 500 mM NaCl and 1 mM TCEP) at 4 °C for 16 h. The digested protein was reloaded onto a HisTrap HP column equilibrated with 50 mM Tris, pH 8.0, 500 mM NaCl and 5 mM β-MCE, and the histidine-HRV tag cleaved DHOase was collected in the flow-through and loaded onto a HiLoad 16/60 Superdex 75 PG gel filtration column that was equilibrated with 50 mM Tris, pH 8.0, 200 mM NaCl and 1 mM TCEP. Protein samples were analyzed by SDS-PAGE, and the final purity was above 90%.

### 3.2. Primary High-Throughput Screen

An HTS of 3000 fragments from the Chembridge fragment library was performed using the optimized DAMO-TSC colorimetric assay. The HTS was completed using a Tecan Freedom EVO 200 liquid handling robot. Final concentrations of 400 µM of each fragment compound were added to 30 µL of 45 nM final concentration of histidine-tagged DHOase in HTS buffer (50 mM Tris, pH 8.3, 0.01% Triton X-100, 0.1 mg/mL BSA and 4 mM TCEP) and incubated for 10 min at room temperature. Then 10 µL of 80 µM final concentration DHO in assay buffer was added, and the mixture was incubated for 30 min at room temperature after shaking for 30 s. Finally, 64 µL of the DAMO-TSC acid mix as previously described was added to quench the reaction. The plates were sealed and incubated in the dark for 16 h at room temperature, and the absorbance was measured at 540 nm. All assays were done in duplicate in transparent 384-well plates. Each plate contained 32 positive and 32 negative controls.

### 3.3. Secondary Counter-Screen by Surface Plasmon Resonance

Purified histidine-tag cleaved DHOase was buffer exchanged to PBS (10 mM phosphate, pH 7.4, 2.7 mM KCl and 137 mM NaCl) for SPR. SPR was performed at 25 °C using a Biacore T200 instrument and a CM5 sensor chip (GE Healthcare). The flow channels were activated by a 1-ethyl-3-(3-dimethylaminopropyl) carbodiimide hydrochloride (EDC)/N-hydroxy succinimide (NHS) mixture. Flow channel 1 was left unmodified as a control. DHOase was diluted in 10 mM sodium acetate (pH 4.0) and immobilized to flow channels 2 and 3 at levels of 8596 RU and 8342 RU, respectively. Fragment solutions at 65 μM and 200 μM were applied to all three channels at a 30 μL/min flow rate. For *K*_D_ determination, fragment solutions at increasing concentrations (0–400 μM at 2-fold dilution) were applied to all three channels at a 30 μL/min flow rate. The SPR binding buffer was PBS-P supplemented with 2% DMSO and 0.5 mM TCEP. All data was referenced with the blank channel RU values, and the sensorgrams were analyzed using Biacore T200 evaluation software v2.0.3. The response units at each concentration was measured during the equilibration phase for the steady-state affinity fittings.

### 3.4. Competition SPR and Analogs

For *K*_D_ determination of analogs, DHOase was immobilized to flow channels 2, 3 and 4 at levels of 10,160 RU, 12,072 RU and 11,358 RU, respectively. 1 mM Ca-asp was additionally added to the binding buffer for a competition SPR. The sensorgrams were analyzed using BiaEvaluation software 2.0.3. All data was referenced to a blank channel RU signal prior to the fitting. The RU difference at each concentration was measured during the binding equilibration phase, and BiaEvaluation software v2.0.3 was used to fit the data to determine *K*_D_ values. The RU values and corresponding concentrations were plotted using the single hyperbolic Function (1), where *y* is the response; *y_max_* is the maximum response, and *x* is the compound concentration.

(1) $$y=\frac{y_{max}\cdot x}{\left(K_{D}+x\right)}$$

### 3.5. Molecular Docking for Compounds ***5***, ***6***, ***8***

Since the residues near the binding site of Ca-asp in DHOase are the same in *S. aureus* and *B. anthracis*, the X-ray crystal structure of *B. anthracis* DHOase complex with Ca-asp (PDB ID: 4YIW [10]) was downloaded from the RCSB protein data bank and prepared by the Protein Preparation Wizard in Schrödinger 2016 [16], including removing crystallographic waters, fixing bond orders, adding hydrogens, assigning partial charges with the OPLS3 force field [17] and minimizing the added hydrogens. The 3D structure of compounds **5**, **6** and **8** were processed by LigPrep module of Schrödinger2016 at pH7.4 [18], and then these ligands were docked into the binding pocket (with a radius of 10 Å around Ca-asp binding site) of the above prepared *B. anthracis* DHOase by Ca-asp, removed using GOLD v5.2.2 [19]. Compounds were set as flexible during the docking process. Standard default settings were used for other parameters. The binding pose for each compound was determined by ChemPLP scoring function by picking the top one [20].

## 4. Conclusions

In summary, a HTS of a small fragment library identified six compounds that inhibited *Sa*DHOase enzyme activity in vitro. SPR was used as a rapid and robust ‘orthogonal’ secondary assay to eliminate false positives from the primary enzymatic screen and perform initial hit characterizations. Mode of inhibition studies of six initial hit compounds and eight additional commercially available analogs with the substrate Ca-asp provided more insights in generating a preliminary SAR map. The 1-benzylpiperidine-4-ol scaffold yielded the most potent, competitive in vitro inhibitors of *Sa*DHOase, with *K*_D_ values 10-times tighter than its substrate Ca-asp in reaching low micromolar ranges, with both competitive SPR and docking studies supporting a competitive mode of inhibition. These results provide novel small compound lead inhibitors and future directions to improve potency further as potential antimicrobial agents against *S. aureus*.

## Figures and Tables

**Table 1:** *K*_D_ Determination of additional analogs containing the main scaffold.

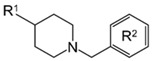			
Compound	R^1^	R^2^	*K*_D_ (µM)
**5**	-OH (*p*)	-4-CH_3_	48 ± 21
**6**	-OH (*p*)	-2,3-Cl	90 ± 4
**7**	-OH (*p*)	-2-CH_3_	41 ± 18
**8**	-OH (*p*)	-2,5-CH_3_	11 ± 5
**9**	-OH (*p*)	-4-CH_2_CH_3_	125 ± 20
**10**	-OH (*p*)	-3-CH_3_	NB ^a^
**11**	-OH (*p*)	-3-CF_3_	NB ^a^
**12**	-OH (*p*)	-4-CF_3_	48 ± 24
**13**	-OH (*m*)	-2,4-CH_3_	77 ± 15
**14**	-CH_2_OH (*m*)	-3-CH_3_	38 ± 5

^a^ NB: No binding.

## Data Availability

All data are presented within manuscript and Supplementary Materials.

## Supplementary Materials

Supplementary Materials are available online https://www.mdpi.com/article/10.3390/ijms22189984/s1.
