# Supplementary material for: Identification of Small Molecule Inhibitors against Staphylococcus aureus Dihydroorotase via HTS
Source: Int J Mol Sci. 2021 Sep 15;22(18):9984. doi: 10.3390/ijms22189984 (PMC8467647; doi:10.3390/ijms22189984)
Supplement: Supplementary file 1 [file ijms-22-09984-s001.zip › ijms-1362757 supplementary.pdf]

Identification of Small Molecule Inhibitors against *Staphylococcus aureus* dihydroorotase via HTS

Amy J. Rice[1,2], Russell P. Pesavento[1,3], Jinhong Ren[1], Isoo Youn[2], Youngjin Kwon[1,4], Chun-Tao Che[2], Michael E. Johnson[1,2,*], Hyun Lee[1,2,4*]

[1] Center for Biomolecular Sciences, College of Pharmacy, University of Illinois at Chicago, Illinois, USA; arice1023@gmail.com; rpesaven@uic.edu; jhren@uic.edu; ykwon35@uic.edu; mjohnson@uic.edu; danielhl@uic.edu

[2] Department of Pharmaceutical Sciences, College of Pharmacy, University of Illinois at Chicago, Illinois, USA; arice1023@gmail.com; iyoun2@uic.edu; chect@uic.edu; mjohnson@uic.edu; danielhl@uic.edu

[3] Department of Oral Biology, College of Dentistry, College of Dentistry, University of Illinois at Chicago, Chicago, Illinois, USA; rpesaven@uic.edu

[4] Biophysics Core at the Research Resource Center, University of Illinois at Chicago, Chicago, Illinois, USA; ykwon35@uic.edu; danielhl@uic.edu

* Correspondence: Hyun Lee: danielhl@uic.edu; 312-355-5292 & Michael E. Johnson: mjohnson@uic.edu; 312-996-9114

Supplementary Data

Confirmed Hit Compound Characterization.

1. **Experimental Methods**

1.1. General Experimental Procedures

Ammonium acetate and 3,5-dinitrobenzoic acid (3,5-DNB) were obtained from Sigma-Aldrich Inc. (St. Louis, MO, USA). HPLC-grade acetonitrile and water came from Fisher Scientific (Waltham, MA, USA). Deuterated dimethyl sulfoxide (DMSO-d_6, 99.9% D) was purchased from Cambridge Isotope Laboratories Inc. (Andover, MA, USA). The HPLC system consisted of Prominence UFLC/ UFLCXR series (Shimadzu, Kyoto, Japan) and a Poroshell 120 column (3.0 mm × 150 mm, 2.7 μm particle size, C18, Agilent, CA, USA). Data analysis was performed utilizing the Shimadzu LabSolutions software. A gastight (25 μL) syringe was purchased from Hamilton Co. (Reno, NV, USA) and 3 mm NMR tubes were obtained from NORELL Inc. (Morganton, NC, USA) for NMR sample preparation. The samples were dried overnight in a Isotemp 280A Vacuum Oven (Fisher Scientific, MA, USA) equipped with VacuuBrand pump (Essex, CT, USA). The quantitative ^{1}H NMR (qHNMR) spectra were recorded on a 400 MHz Bruker AVIII HD NMR spectrometer equipped with a 5 mm room temperature SmartProbe™ (Karlsruhe, Germany). NMR analysis was performed in Mnova 14.0.2 software (Mestrelab Research S. L., Santiago de Compostela, Spain).

1.2. HPLC Analysis

The mobile phase was (A) 20 mM ammonium acetate buffer and (B) acetonitrile. The flow rate was 0.7 mL/min and the injection volume was 5 μL. The column temperature was kept at 40 °C and the detection wavelength was 210 nm. The mobile phase gradient was as follows: 5% B in 2

min, 5–100% B in 2–13 min, held isocratic at 100% B up to 14 min and reconditioning at 5% B up to 20 min.

1.3. Quantitative NMR Analysis

As an internal calibrant, 3.20 mg of 3,5-DNB was dissolved in 500 µL of DMSO-d_6 and 50 µL of this stock solution was added to each NMR sample to meet 170 µL of total volume. The samples were dried at 40 °C overnight, and then dissolved in 170 µL of DMSO-d_6 and transferred into 3 mm NMR tubes. The parameters for qHNMR experiment were a relaxation delay (D1) of 20 s, a calibrated 90 ° pulse (P1), number of scans (ns) of 32, receiver gain (RG) of 32 and an acquisition time of 4.0 s. NMR data was processed with zero-filling of the 64k FID to 256k real data, a Lorentzian-Gaussian window function (exponential factor -0.30, Gaussian factor 0.05) and a base line correction (third order polynomial). The purity of the target compounds was quantified by absolute qHNMR method with an internal standard, 3,5-DNB.[1]

2. Results

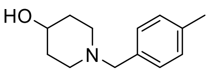

5

Chemical Formula: $C_{13}H_{19}NO$
Molecular Weight: 205.30

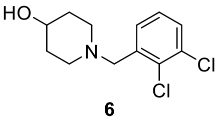

6

Chemical Formula: $C_{12}H_{15}Cl_2NO$
Molecular Weight: 260.16

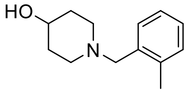

7

Chemical Formula: $C_{13}H_{19}NO$
Molecular Weight: 205.30

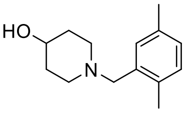

8

Chemical Formula: $C_{14}H_{21}NO$
Molecular Weight: 219.33

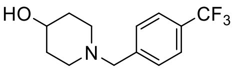

12

Chemical Formula: $C_{13}H_{16}F_3NO$
Molecular Weight: 259.27

2.1. HPLC Analysis

The purities of **5**,**6**,**7**,**8** and **12** were 99.82, 99.22, 99.53, 98.54 and 99.17 %, respectively.

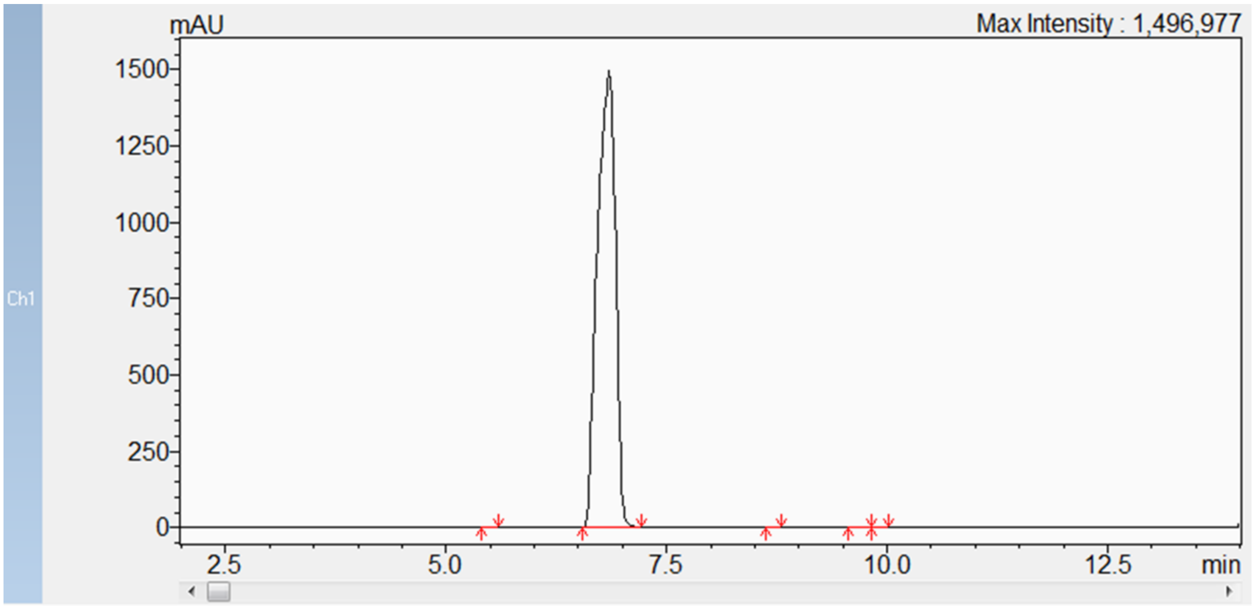

Results View - Peak Table

Peak Table | Compound | Group | Calibration Curve

Peak#	Ret. Time	Area	Height	Conc.	Area%
1	5.474	5988	1773	0.028	0.028
2	6.850	21554953	1494953	99.818	99.818
3	8.718	14024	2681	0.065	0.065
4	9.700	16386	2524	0.076	0.076
5	9.882	2989	445	0.014	0.014
Total		21594340	1502376	100.000	100.000

Fig. S1. HPLC-UV chromatogram and its purity table for **5**.

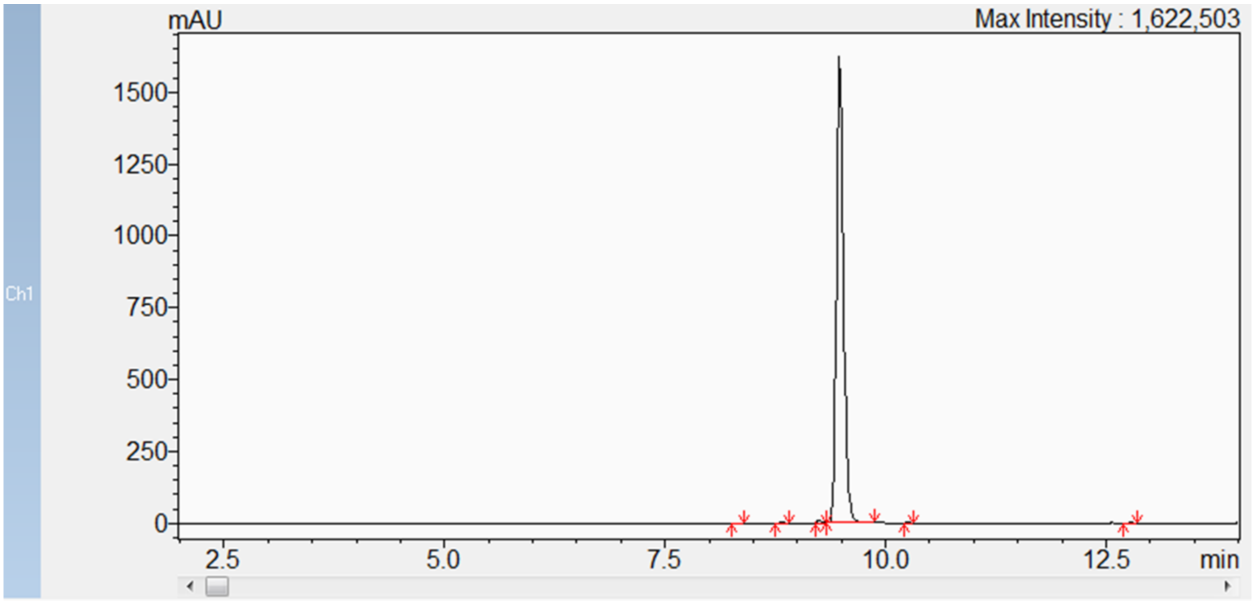

Results View - Peak Table

Peak Table | Compound | Group | Calibration Curve

Peak#	Ret. Time	Area	Height	Conc.	Area%
1	8.306	10741	1853	0.119	0.119
2	8.830	6001	1490	0.067	0.067
3	9.242	41427	8868	0.460	0.460
4	9.478	8927104	1620553	99.221	99.221
5	10.250	4869	1504	0.054	0.054
6	12.777	7011	2345	0.078	0.078
Total		8997153	1636612	100.000	100.000

Fig. S2. HPLC-UV chromatogram and its purity table for **6**.

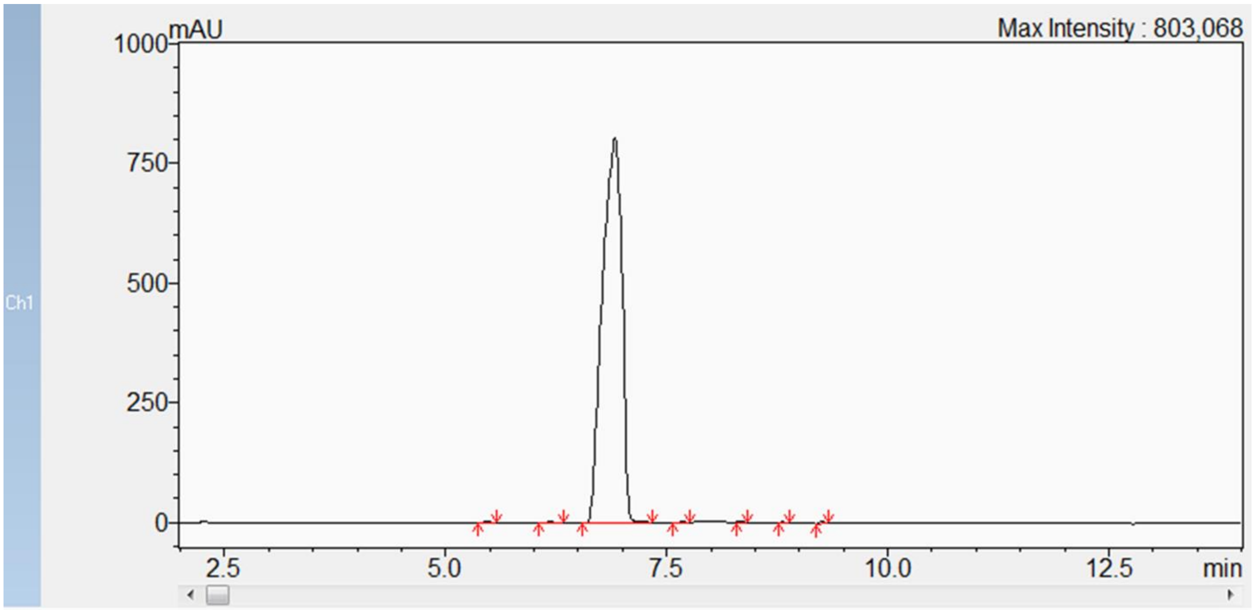

Results View - Peak Table

Peak Table | Compound | Group | Calibration Curve

Peak#	Ret. Time	Area	Height	Conc.	Area%
1	5.464	12870	2689	0.104	0.104
2	6.185	9601	1767	0.078	0.078
3	6.918	12304390	803943	99.529	99.529
4	7.687	6794	1306	0.055	0.055
5	8.308	5424	1599	0.044	0.044
6	8.813	5483	1656	0.044	0.044
7	9.257	18000	4544	0.146	0.146
Total		12362562	817504	100.000	100.000

Fig. S3. HPLC-UV chromatogram and its purity table for **7**.

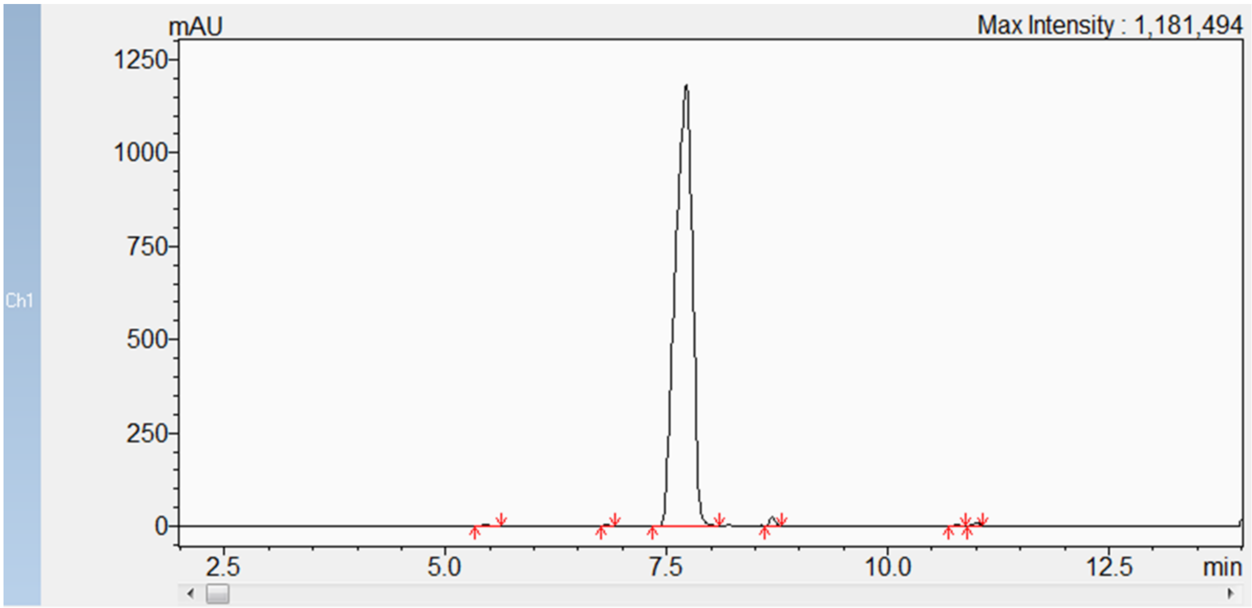

Results View - Peak Table

Peak Table | Compound | Group | Calibration Curve

Peak#	Ret. Time	Area	Height	Conc.	Area%
1	5.456	20658	5473	0.128	0.128
2	6.815	23194	5082	0.143	0.143
3	7.723	15942711	1181314	98.539	98.539
4	8.695	124595	26242	0.770	0.770
5	10.784	20766	3309	0.128	0.128
6	11.003	47124	9762	0.291	0.291
Total		16179048	1231181	100.000	100.000

Fig. S4. HPLC-UV chromatogram and its purity table for **8**.

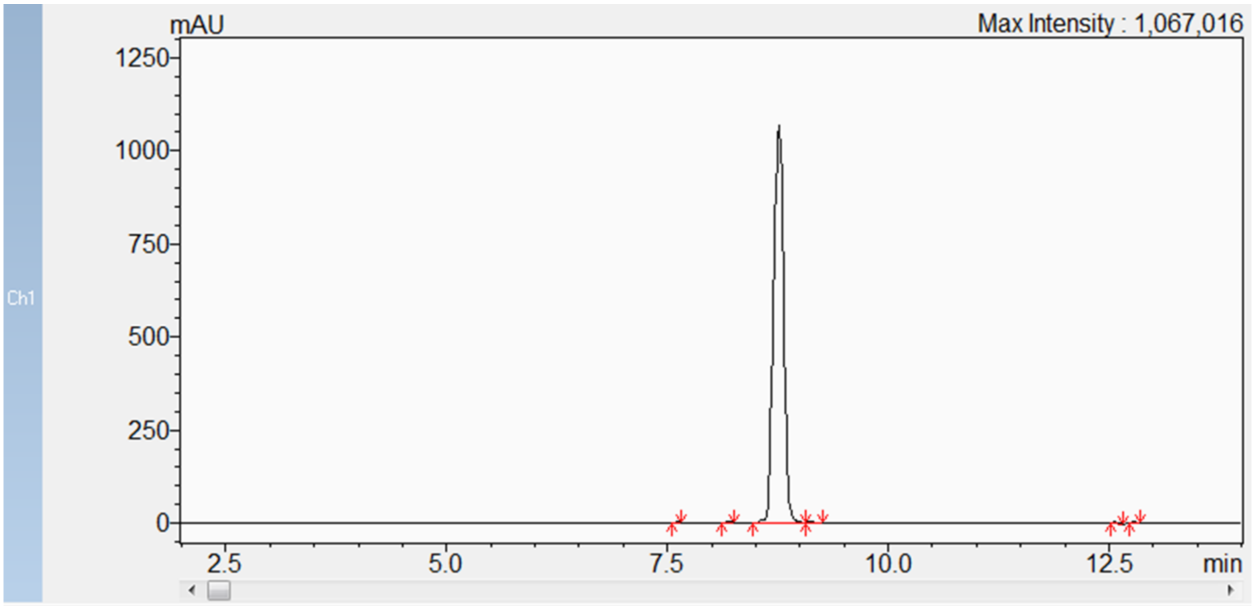

Results View - Peak Table

Peak Table | Compound | Group | Calibration Curve

Peak#	Ret. Time	Area	Height	Conc.	Area%
1	7.624	6807	3432	0.078	0.078
2	8.202	14625	4688	0.167	0.167
3	8.765	8682483	1066075	99.174	99.174
4	9.117	15879	3813	0.181	0.181
5	12.569	16333	3377	0.187	0.187
6	12.777	18658	4956	0.213	0.213
Total		8754785	1086341	100.000	100.000

Fig. S5. HPLC-UV chromatogram and its purity table for **12**.

2.2. Quantitative ^{1}H NMR Analysis

The peak integration was achieved in the aromatic regions in the spectra. The purities of **6**,**7** and **8** were 96.77, 96.73 and 98.50 %, respectively.

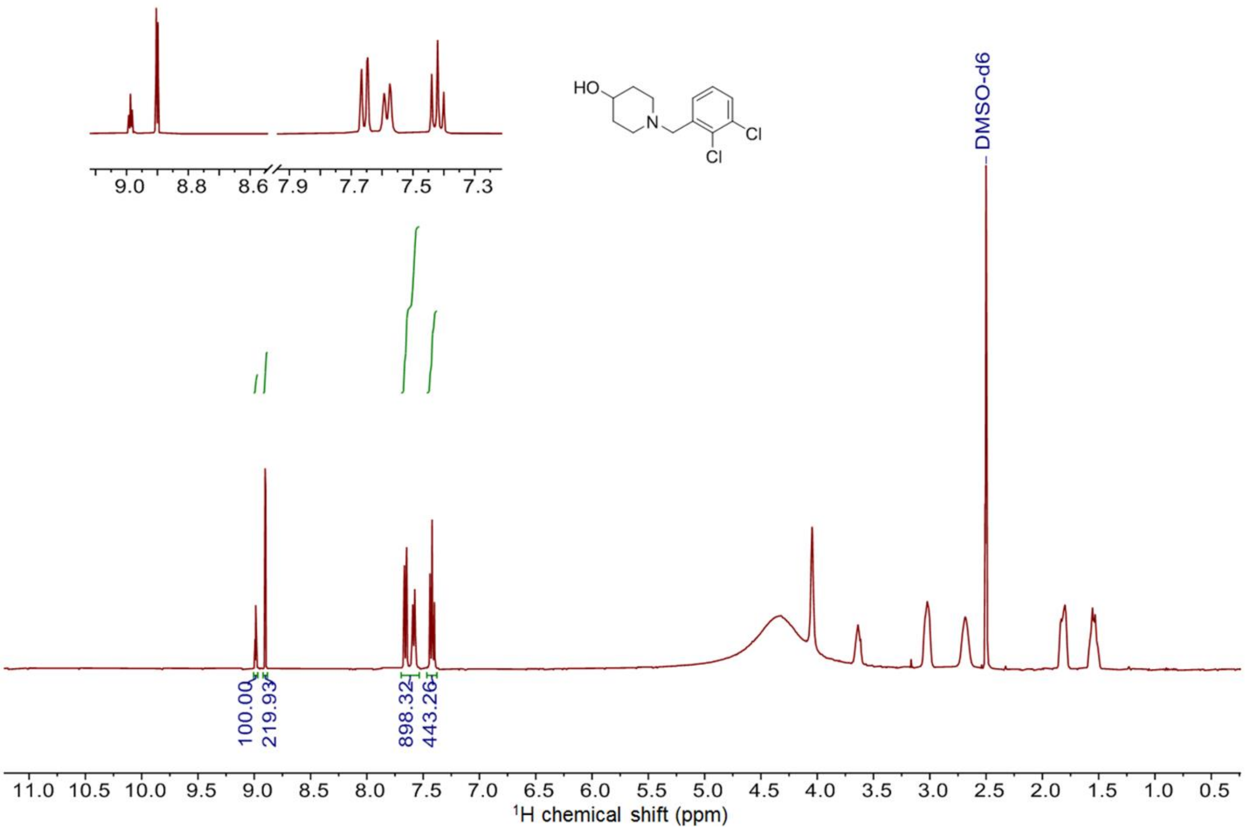

Fig. S6. Quantitative ^{1}H NMR spectrum for **6** (DMSO-d_6, 400MHz). The purity of the compound was 96.77 %.

Absolute Method w/Internal Calibration (IC)

Sample Containing One Internal Calibrant

$$P\,[\%] = \frac{n_{IC} \cdot Int_t \cdot MW_t \cdot m_{IC}}{n_t \cdot Int_{IC} \cdot MW_{IC} \cdot m_s} \cdot P_{IC}$$

			Calculated Fiels in BLUE - Do Not Change!
**Start Here--->**		**A - Data Entry**	Enter data in yellow fields ONLY
		**B - Result**	**The Purity of the 5256888 sample is 96.77%**
		**C - Note**	Consider the weighing precision when determining the number of decimal places for the reported purity
**Parameter**	**STEP**	**Variable or *Index***	**Value**
**Target Analyte**			
Name		*t*	**5256888**
Molecular Weight	**4**	MW_t	260.16
			2.95
Integral Value	**2**	Int_t	446.21
Number of Protons Giving Rise to Integral	**2**	n_t	1
**Sample**			
Exact Weight (mass) of Sample	**1**	m_s	**1.68**
**Internal Calibrant**			
Name		*IC*	**3,5-DNB**
Molecular Weight	**4**	MW_{IC}	**212.12**
Exact Weight (Mass) of Calibrant in Sample	**1**	m_{IC}	**0.320**
Purity of the Internal Calibrant	**1**	P_{IC} (fraction of 1)	**0.990**
Integral Value	**3**	Int_{IC}	**319.93**
Number of Protons Giving Rise to Integral	**3**	n_{IC}	**3**
**Purity**			
Calculated Purity	**5**	P	96.77

Please read the disclaimer

**STEP 2:**	**Target Analyte Integrals [Single or Multiple Integrals]**							
**Calculation of Average Integrals for 1H**								
**Note**: leave YELLOW fields blank for unused values; do not use zero!								
<--St.Dev. of Integrals	Int# 1	Int# 2	Int# 3	Int# 4	Int# 5	Int# 6	Int# 7	Int# 8
<--Average Int per 1H	**898.32**	**443.26**						
# of Hs-->	**2**	**1**						
Integral per 1H-->	449.16	443.26						
100	219.93							

Fig. S7. The calculation sheet for purity assessment with quantitative ^{1}H NMR method for **6**.

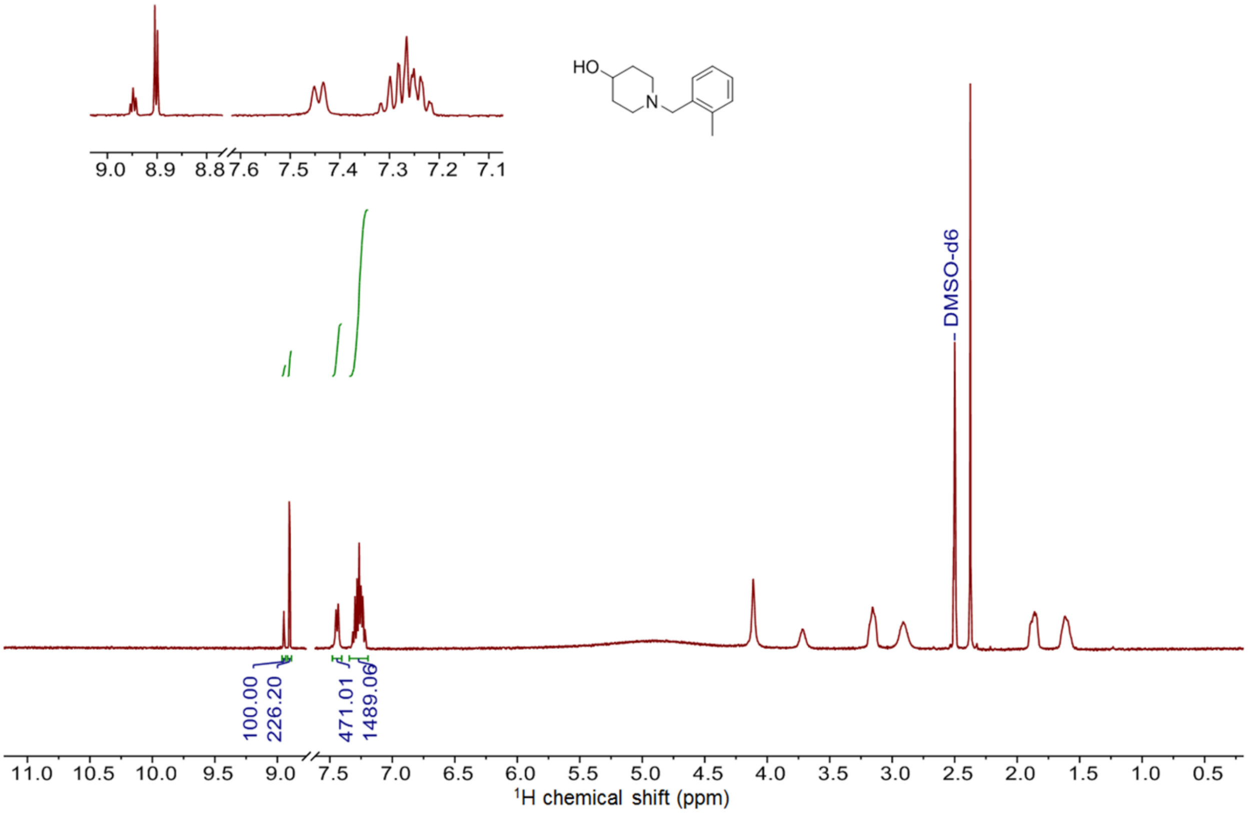

Fig. S8. Quantitative ^{1}H NMR spectrum for **7** (DMSO-d_6, 400MHz). The purity of the compound was 96.73 %.

**Absolute Method w/Internal Calibration (IC)**				Please read the disclaimer								
**Sample Containing One Internal Calibrant**			Calculated Fiels in BLUE - Do Not Change!									
**Start Here--->**		**A - Data Entry**	Enter data in yellow fields ONLY									
$P\,[\%] = \frac{n_{IC} \cdot Int_t \cdot MW_t \cdot m_{IC}}{n_t \cdot Int_{IC} \cdot MW_{IC} \cdot m_s} \cdot P_{IC}$		**B - Result**	**The Purity of the 5264100 sample is 96.73%**									
		**C - Note**	Consider the weighing precision when determining the number of decimal places for the reported purity									
**Parameter**	**STEP**	**Variable or *Index***	**Value**	**STEP 2:**	**Target Analyte Integrals [Single or Multiple Integrals]**							
**Target Analyte**				**Calculation of Average Integrals for 1H**								
Name		*t*	**5264100**	**Note**: leave YELLOW fields blank for unused values; do not use zero!								
Molecular Weight	**4**	MW_t	205.3									
			12.67	<--St.Dev. of Integrals	Int# 1	Int# 2	Int# 3	Int# 4	Int# 5	Int# 6	Int# 7	Int# 8
Integral Value	**2**	Int_t	483.68	<--Average Int per 1H	**471.01**	**1489.06**						
Number of Protons Giving Rise to Integral	**2**	n_t	1	# of Hs-->	**1**	**3**						
				Integral per 1H-->	471.01	496.35						
**Sample**												
Exact Weight (mass) of Sample	**1**	m_s	**1.41**									
**Internal Calibrant**												
Name		*IC*	**3,5-DNB**									
Molecular Weight	**4**	MW_{IC}	**212.12**									
Exact Weight (Mass) of Calibrant in Sample	**1**	m_{IC}	**0.320**									
Purity of the Internal Calibrant	**1**	P_{IC} (fraction of 1)	**0.990**									
Integral Value	**3**	Int_{IC}	**326.20**	100	226.2							
Number of Protons Giving Rise to Integral	**3**	n_{IC}	**3**									
**Purity**												
Calculated Purity	**5**	P	96.73									

Fig. S9. The calculation sheet for purity assessment with quantitative ^{1}H NMR method for **7**.

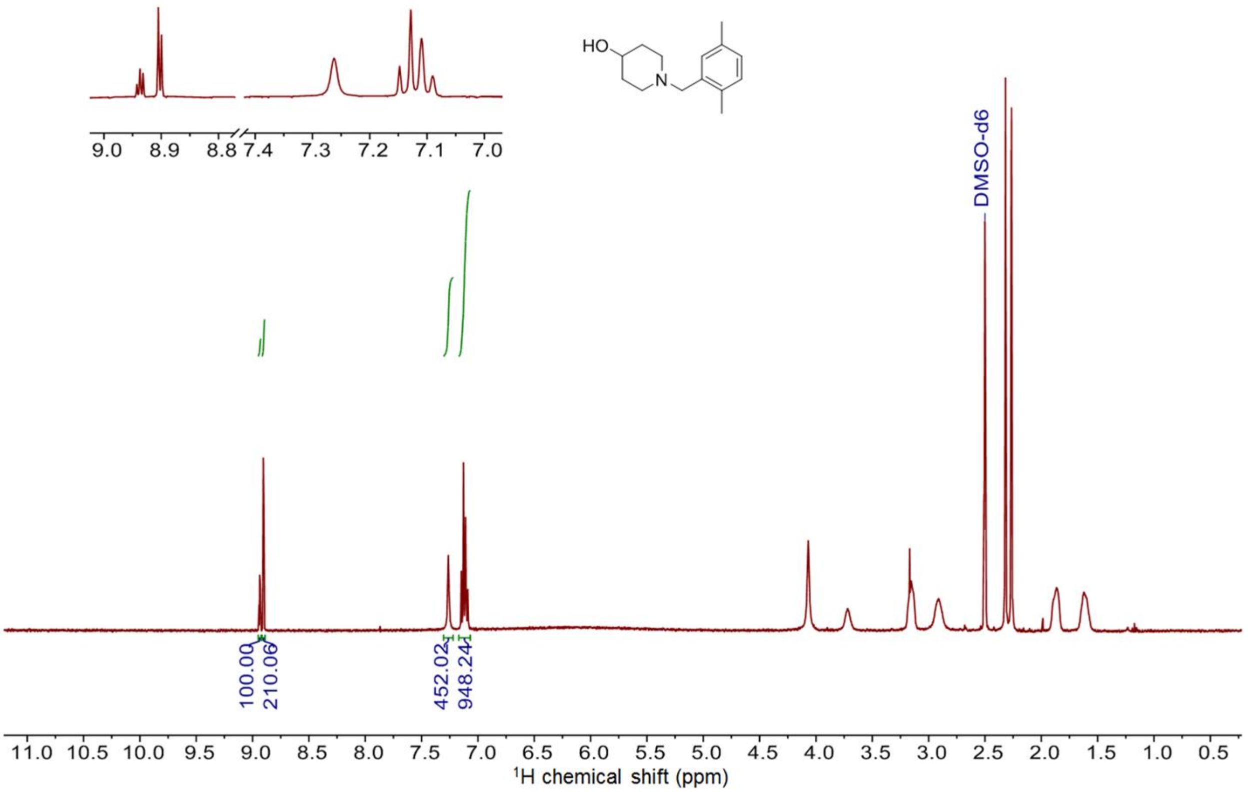

Fig. S10. Quantitative ^{1}H NMR spectrum for **8** (DMSO-d_6, 400MHz). The purity of the compound was 98.50 %.

**Absolute Method w/Internal Calibration (IC)**				Please read the disclaimer								
**Sample Containing One Internal Calibrant**			Calculated Fiels in BLUE - Do Not Change!									
**Start Here--->**		**A - Data Entry**	Enter data in yellow fields ONLY									
$P\,[\%] = \frac{n_{IC} \cdot Int_t \cdot MW_t \cdot m_{IC}}{n_t \cdot Int_{IC} \cdot MW_{IC} \cdot m_s} \cdot P_{IC}$		**B - Result**	**The Purity of the 5260449 sample is 98.50%**									
		**C - Note**	Consider the weighing precision when determining the number of decimal places for the reported purity									
**Parameter**	**STEP**	**Variable or *Index***	**Value**	**STEP 2:**		**Target Analyte Integrals [Single or Multiple Integrals]**						
**Target Analyte**				**Calculation of Average Integrals for 1H**								
Name		*t*	**5260449**	**Note:** leave YELLOW fields blank for unused values; do not use zero!								
Molecular Weight	**4**	MW_t	219.32									
			11.05	<--St.Dev. of Integrals	Int# 1	Int# 2	Int# 3	Int# 4	Int# 5	Int# 6	Int# 7	Int# 8
Integral Value	**2**	Int_t	463.07	<--Average Int per 1H	**452.02**	**948.24**						
Number of Protons Giving Rise to Integral	**2**	n_t	1	# of Hs-->	**1**	**2**						
				Integral per 1H-->	452.02	474.12						
**Sample**												
Exact Weight (mass) of Sample	**1**	m_s	**1.49**									
**Internal Calibrant**												
Name		*IC*	**3,5-DNB**									
Molecular Weight	**4**	MW_{IC}	**212.12**									
Exact Weight (Mass) of Calibrant in Sample	**1**	m_{IC}	**0.320**									
Purity of the Internal Calibrant	**1**	P_{IC} (fraction of 1)	**0.990**									
Integral Value	**3**	Int_{IC}	**310.06**	100	210.06							
Number of Protons Giving Rise to Integral	**3**	n_{IC}	**3**									
**Purity**												
Calculated Purity	**5**	P	98.50									

Fig. S11. The calculation sheet for purity assessment with quantitative ^{1}H NMR method for **8**.

1. Pauli GF, Chen S-N, Simmler C, et al. Importance of purity evaluation and the potential of quantitative ^{1}H NMR as a purity assay: miniperspective. *Journal of Medicinal Chemistry*. 2014;57(22): 9220-9231.
